# Inoculation effect of *Pseudomonas* sp. TF716 on N_2_O emissions during rhizoremediation of diesel-contaminated soil

**DOI:** 10.1038/s41598-022-17356-z

**Published:** 2022-07-29

**Authors:** Ji-Yoon Kim, Kyung-Suk Cho

**Affiliations:** Department of Environmental Science and Engineering, Ewha Womans University, Seoul, 03760 Republic of Korea

**Keywords:** Environmental sciences, Solid Earth sciences

## Abstract

The demand for rhizoremediation technology that can minimize greenhouse gas emissions while effectively removing pollutants in order to mitigate climate change has increased. The inoculation effect of N_2_O-reducing *Pseudomonas* sp. TF716 on N_2_O emissions and on remediation performance during the rhizoremediation of diesel-contaminated soil planted with tall fescue (*Festuca arundinacea*) or maize (*Zea mays*) was investigated. *Pseudomonas* sp. TF716 was isolated from the rhizosphere soil of tall fescue. The maximum N_2_O reduction rate of TF716 was 18.9 mmol N_2_O g dry cells^−1^ h^−1^, which is superior to the rates for previously reported *Pseudomonas* spp. When *Pseudomonas* sp. TF716 was added to diesel-contaminated soil planted with tall fescue, the soil N_2_O-reduction potential was 2.88 times higher than that of soil with no inoculation during the initial period (0–19 d), and 1.08–1.13 times higher thereafter. However, there was no enhancement in the N_2_O-reduction potential for the soil planted with maize following inoculation with strain TF716. In addition, TF716 inoculation did not significantly affect diesel degradation during rhizoremediation, suggesting that the activity of those microorganisms involved in diesel degradation was unaffected by TF716 treatment. Analysis of the dynamics of the bacterial genera associated with N_2_O reduction showed that *Pseudomonas* had the highest relative abundance during the rhizoremediation of diesel-contaminated soil planted with tall fescue and treated with strain TF716. Overall, these results suggest that N_2_O emissions during the rhizoremediation of diesel-contaminated soil using tall fescue can be reduced with the addition of *Pseudomonas* sp. TF716.

## Introduction

Petroleum oil is the most widely used energy source globally, and petroleum hydrocarbons (PHs) can leak into the soil and groundwater during its storage, transportation, and use, threatening the environment^1–3^. PHs that contaminate the soil not only disturb the ecological balance but also damage the surrounding landscape and emit odors, causing aesthetic and economic damage^4,5^. Of the various soil remediation technologies that have been proposed, rhizoremediation is particularly promising because it can accelerate the biodegradation of PHs by taking advantage of the close interaction between plant roots and rhizospheric microorganisms^1^.

In the rhizoremediation of PH-contaminated soil, nitrogen sources are typically added to improve the biodegradation efficiency, which may result in nitrous oxide (N_2_O) emissions^6^. Ammonium in the soil is typically nitrified and then denitrified by microorganisms and emitted into the atmosphere in the form of N_2_. However, if the reduction of N_2_O, an intermediate metabolite, to N_2_ does not occur, N_2_O may be released^6–8^. N_2_O is a significant greenhouse gas with a global warming potential (GWP) that is about 298 times higher than that of CO_2_^9^. Once discharged into the atmosphere, N_2_O has a long residence time of about 121 years^9^ and is a precursor of nitric oxide (NO), which destroys the ozone layer in the stratosphere via photochemical reactions^10,11^.

As part of the goal to remove environmental pollutants as a response to climate change, it is necessary to minimize N_2_O emissions during the remediation of PH-contaminated soil. A useful solution in this regard is to actively use bacteria that can reduce N_2_O to N_2_. Therefore, in the present study, the effect of a N_2_O-reducing bacterium on N_2_O emissions during the rhizoremediation of PH-contaminated soil was evaluated. A bacterial isolate with excellent N_2_O-reduction ability was extracted from an enriched broth that employed rhizosphere soil as the inoculum source, and N_2_O was supplied as a final electron acceptor. The inoculation effect of the isolated bacterium on the N_2_O-reduction potential of soil samples was evaluated for the rhizoremediation of PH-contaminated soil planted with tall fescue (*Festuca arundinacea*) or maize (*Zea mays*). In addition, the inoculation effect of the isolate on the PH-removal efficiency, plant growth, and soil bacterial community associated with N_2_O reduction was also investigated.

## Materials and methods

### Isolation and identification of a N_2_O-reducing bacterium

A soil sample (2 g) obtained from the rhizosphere of tall fescue (*F. arundinacea*) was suspended in 200 mL of a mineral medium containing KH_2_PO_4_ (169.7 mg L^−1^), MgSO_4_·7H_2_O (751.1 mg L^−1^), CaCl·2H_2_O (451.6 mg L^−1^), EDTA (5.0 mg L^−1^), FeSO_4_·7H_2_O (5.0 mg L^−1^), and 1 mg L^−1^ of a trace element solution. The trace element solution contained ZnSO_4_·7H_2_O (0.43 mg L^−1^), CoCl_2_·6H_2_O (0.24 mg L^−1^), MnCl_2_·4H_2_O (0.99 mg L^−1^), CuSO_4_·5H_2_O (0.25 mg L^−1^), Na_2_MoO_4_·2H_2_O (0.22 mg L^−1^), NiCl_2_·6H_2_O (0.19 mg L^−1^), and Na_2_SeO_4_ (0.21 mg L^−1^). The pH of the medium was adjusted to 7 with 1 M NaOH and 1 M HCl solutions. The soil suspension was added to a 1.2-L serum bottle and then sealed with a butyl rubber stopper after purging with N_2_ gas to maintain anaerobic conditions. Sterile stock solutions of glucose and sodium acetate as a carbon source were added to the bottle with a syringe to a final concentration of 100 mg L^−1^ each based on the chemical oxygen demand (COD). N_2_O gas (99%; Dong-A Specialty Gases Co., Seoul, Republic of Korea) was injected into the bottle via a syringe to adjust the final headspace concentration to 1000 ppm (v/v). The serum bottle was incubated at 30 °C under agitation at 120 rpm. The N_2_O concentration was monitored periodically using a gas chromatograph (Agilent Technologies, Santa Clara, CA, USA) equipped with a micro-electron capture detector^8^. When the N_2_O concentration in the headspace of the bottle decreased below 10 ppm, additional N_2_O gas was injected into the bottle to a headspace concentration of 1000 ppm (v/v). The injection of N_2_O gas was repeated in this way a further six times. Following this, 40 ml of the culture was transferred to a serum bottle containing 160 ml of the fresh mineral medium. The transferred culture was incubated under the same conditions as described above. The N_2_O-reducing consortium of bacteria was obtained after the transfer process had been repeated five times in the same manner as described above.

The N_2_O-reducing consortium was diluted serially with sterile distilled water and spread onto Difco™ LB agar plates. The LB agar medium (pH 7.0) contained tryptone (10 g L^−1^), yeast extract (5 g L^−1^), NaCl (10 g L^−1^), and agar (15 g L^−1^). The plates were incubated at 30 °C for 48 h. Each colony was then transferred to a fresh LB agar plate and incubated under the same conditions. This process was repeated until eight pure colonies had been obtained.

The N_2_O-reduction activity of the eight isolates was then compared. The isolates were incubated in 10 mL of LB broth at 30 °C for 24 h at 120 rpm under aerobic conditions. Each culture broth was centrifuged, and the obtained cell pellet was rinsed with distilled water and used to prepare a cell suspension with an optical density (OD_600nm_) of 1.0 in a mineral medium containing NaCl (8 g L^−1^), KCl (0.2 g L^−1^), Na_2_HPO_4_ (1.44 g L^−1^), and KH_2_PO_4_ (0.24 g L^−1^) (pH 7.0). The optical density was measured spectrophotometrically at 600 nm using a Libra S22 spectrophotometer (Biochrom, Cambridge, UK). A 1 mL aliquot of each cell suspension was added to a 120-mL serum bottle containing 9 mL of the fresh mineral medium. The bottle was sealed with a butyl rubber stopper after purging with N_2_ gas, and the carbon source was added to the bottle with a syringe to a final concentration of 100 mg L^−1^ based on the COD. N_2_O gas was injected into each bottle to a concentration of 1000 ppm, and the N_2_O concentration in the sample headspace was monitored over time using a gas chromatograph. Each experiment was performed in triplicate. The isolate with the highest activity was labeled isolate TF716.

To identity isolate TF716, a TF716 colony was mixed with 15 μL of sterilized water and treated for 3 min in a heating block at 95 °C. After the cellular debris was removed via centrifugation (7500 × *g*), the DNA sample in the obtained supernatant was used as a template for PCR. To target the 16S rRNA gene, 340F and 805R primers were used for amplification^8^. The PCR products were sequenced by Macrogen (Seoul, Republic of Korea) and the resulting sequence was analyzed using the Basic Local Alignment Search Tool (BLAST) developed by the National Center for Biotechnology Information (NCBI). A molecular phylogenetic tree was constructed with the 16S rRNA sequences of isolate TF716 and known N_2_O- and oil-degrading bacteria using MEGA software (version 11, www.megasoftware.net) and a neighbor-joining algorithm.

### Characterization of the N_2_O-reduction activity of isolate TF716

The effects of the N_2_O concentration and root exudate on N_2_O reduction by strain TF716 were characterized. An aliquot of a cell suspension (1 mL) with an OD_600_ of 1.0 prepared in the manner described above was added to a 120-ml serum bottle containing 9 mL of a mineral medium. The carbon solution was injected into the bottle after purging and sealing in the same manner as described above. To determine the effect of the N_2_O concentration on the N_2_O-reduction activity of strain TF716, N_2_O gas was injected into the bottle to a concentration of 200, 500, 750, 1000, or 1500 ppm.

The root exudate of tall fescue (*F. arundinacea*) and maize (*Z. mays*) was prepared in a similar manner to that described in a previous paper^12^. Tall fescue and maize, grown in non-contaminated soil, was carefully sampled, and the soil adhering loosely to the root removed by shaking the plant. After washing with distilled water (DW) several times, 10 g of each root sample was added into 100 ml of sterilized DW, and mixed for 2 h at 50 °C. After cooling each mixed solution at room temperature, the mixed solution was filtered with 0.45 µm filter. Each filtered solution was used as root exudate. The COD of the root exudate for tall fescue and maize was 750 and 780 mg L^−1^, respectively. The compounds in the mineral medium were also added to the root exudate at the same concentrations as in the mineral medium to produce the root exudate medium. The cell suspension was added to a 120-ml serum bottle containing 9 mL of the root exudate medium. For the control, the carbon source was added to all conditions. N_2_O gas was injected into each bottle to a final concentration of 1000 ppm. All experiments were performed at 30 °C with 120 rpm in triplicate. The N_2_O reduction rate for each experimental condition was calculated as described in a previous paper^8^. The maximum N_2_O reduction rate (*V*_*max*_) and saturation constant (*K*_*m*_) were determined using Lineweaver–Burk plots (Park et al., 2020). Cell concentrations were determined from the relationship between the optical density measured at 600 nm and the dry cell weight (DCW, g). To determine the dry cell weight, the cells in the cell suspension were harvested via centrifugation (7500 × *g*) and dried at 70 °C for 24 h.

### Pot experiment

A pot experiment was conducted to evaluate the inoculation effect of the isolate TF716 on the rhizoremediation performance of diesel-contaminated soil planted with tall fescue or maize. Soil was collected from a garden on the rooftop of the New Engineering Building at Ewha Womans University, Seoul, Republic of Korea (37°57′ N, 126°95′ E). The soil texture was loamy sand. After sieving the soil with a 2-mm sieve to remove weeds and stones, the soil was artificially contaminated with diesel at initial concentrations of 10,000 mg-diesel kg-soil^−1^. The contaminated soil was then stored in a shaded location on the rooftop for seven days and mixed uniformly once a day. To provide N and P, compost was added to the contaminated soil at a 1:19 (w/w) ratio. The compost was purchased from a commercial vendor (Seokgang Green Fertilizer Inc., Icheon, Republic of Korea) and consisted of fermented swine manure (40%), sawdust (49%), cow manure (10%), and the bacterial inoculum (1%). The NH_4_^+^–N, NO_3_^–^N, and total P concentrations of the contaminated soil amended with compost were 12.2, 15.7, and 1195.2 mg kg-soil^−1^, respectively. The organic matter content and pH were 27.1% and 6.8, respectively. The cell suspension of isolate TF716, which was prepared by cultivating the cells in the LB medium as described above, followed by harvesting and rinsing, was added to the soil samples at a cell concentration of 2 × 10^6^ CFU∙kg-soil^−1^.

A drainer and coarse sand were first placed on the bottom of each pot (diameter of 180 mm; height of 150 mm), and 3 kg of the contaminated soil with or without isolate TF716 was then added to each pot. Ten tall fescue or five maize seedlings were planted in each pot. The pot experiment was conducted in triplicate. The tall fescue seedlings were cultivated from seed for 45 days in a garden on the rooftop of the New Engineering Building at Ewha Womans University. Maize seedlings were purchased (Mojong 114, Gyeonggi-do, Republic of Korea), and cultivated for 45 days in the garden. The pot experiment was conducted on the rooftop of the New Engineering Building, Ewha Womans University for 59 days (May 28 to July 26, 2021). The pot soil was watered twice a week to maintain an average water content of about 28% during the experimental period. During the pot experiment, the pH of the soil ranged from 6.5 to 7.2, and there was no significant difference in the soil pH between the experimental conditions (data not shown).

### Inoculation effect of isolate TF716 on the N_2_O-reduction potential of the soil and rhizoremediation performance

To evaluate the inoculation effect of isolate TF716 on the N_2_O-reduction potential of the soil during the rhizoremediation of diesel-contaminated soil, rhizosphere soil samples were taken randomly from each pot on days 0, 19, 40, and 59. After air-drying at room temperature, 10 g of each soil sample was added to a 600-mL serum bottle containing 30 ml of the mineral medium. The N_2_O reduction potential was evaluated in the same manner as described previously^13^.

To analyze the residual diesel concentrations in the soil sampled on days 19 and 59, the collected soil was freeze-dried, and 3 g of each sample was consequently added to individual test tubes, after which 10 mL of hexane–acetone (1:1, v/v) solution was added as the solvent for extraction. The diesel concentration was measured using the same method and gas chromatograph (GC 6980 N system, Agilent Technologies, CA, USA) as described in a previous study^13^. On day 59, the roots of the tall fescue and maize were sampled after shaking the rhizosphere soil away from the roots. The roots were then dried in an oven at 70 °C for 72 h, and then the dry weight of the roots was measured.

To characterize the dynamics of the bacterial community associated with N_2_O reduction during rhizoremediation, the bacterial metagenome in the soil was analyzed using *nos*Z primer sets targeting the N_2_O reductase gene. Genomic DNA from the air-dried soil samples was extracted using a NucleoSpin® Soil Kit (Macherey–Nagel GmbH, Düren, Germany) and a BeadBeater-8 system (BioSpec Products Inc., Bartlesville, OK, USA) according to the manufacturers’ instructions. The genomic DNA samples were eluted in 50 µL of elution buffer and quantified using a SpectraMax QuickDrop spectrophotometer (Molecular Devices, San Jose, CA, USA). The extracted genomic DNA samples were then stored at − 23 °C before use. PCR was performed based on a primer set of amplicon-*nos*ZIF (5’-*TCGTCGGCAGCGTCAGATGTGTATAAGAGACAGWCSYTGTTCMTCGACAGCCAG*-3’) and amplicon-*nos*ZIR (5’-*GTCTCGTGGGCTCGGAGATGTGTATAAGAGACAGATGTCGATCARCTGVKCRTTYTC*-3’)^14^. The underlined sequences are pre-adapters, the sequences in bold are sequencing primer sequences, and the sequences in italics are the specific locus primers. The PCR steps were conducted as described in previous reports^13,14^. The sequences were analyzed via an Illumina MiSeq sequencing platform (Illumina Inc.) by Macrogen Inc. (Seoul, Republic of Korea). The sequence reads were analyzed using QIIME 1.9 by Macrogen Inc.^15^. Sequences outside of the target base pairs were removed using Fast Length Adjustment of Short Reads (FLASH) 1.2.11^16^. Ambiguous and chimeric sequences were then removed and sequences were classified into operational taxonomic units (OTUs) at a 97% similarity using the CD-HIT-OTU program^17^. The taxonomy for each OTU was assigned based on the NCBI 16S microbial database.

### Statistical analysis

Microsoft Excel 2013 (Microsoft Co., Redmond, WA, USA) was employed to conduct *t*-tests and multiple comparisons with a *P-*value of 0.05 used to indicate a significant difference. Pearson correlations between parameters were also calculated using R (www.rstudio.com).

## Results and discussion

### N_2_O reduction activity of isolate TF716

**Figure 1:**
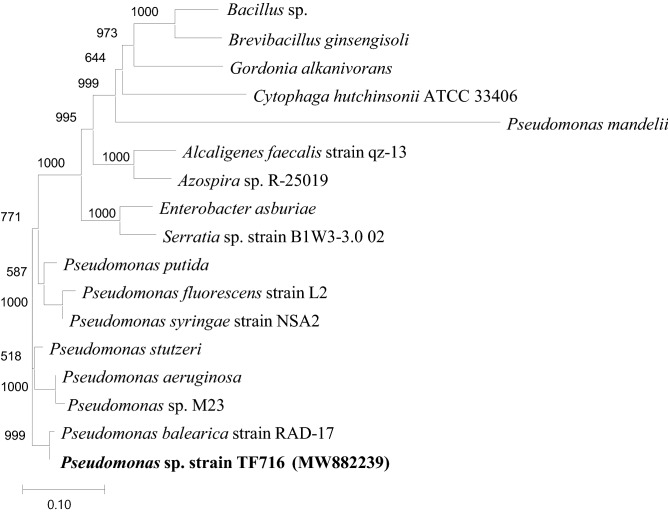
Phylogenetic relationships between *Pseudomonas* sp. TF716 and known N_2_O-reducing bacteria. A neighbor-joining algorithm was used to construct the tree. The scale bar represents 0.1 expected substitutions per site.

Strain TF716, which was isolated from the N_2_O-reducing consortium, was identified as *Pseudomonas* sp. (Fig. 1), which is the first N_2_O-reducing rhizobacterium to be isolated from the rhizosphere soil of tall fescue. Other N_2_O-reducing *Pseudomonas* spp. have been isolated from soil^18,19^, biofilters for treating landfill leachate^20^, and wastewater treatment plants^21^. *Azospira* spp. are N_2_O reducers that have also been isolated from wastewater treatment processes^8,19,22^. Other N_2_O-reducing bacteria that have been reported include *Bacillus amyloliquefaciens*^23^ and *Ralstonia solanacearum*^24^ isolated from soil*, Paracoccus pantotrophus*^25^ and *Alicyliphilus* sp.^22^ isolated from wastewater treatment processes, and *Aeromicrobium massiliense*^26^ isolated from human fecal manure. Usyskin-Tonne et al.^27^ also isolated the N_2_O-reducing bacteria *Agrobacterium/Rhizobium* sp., *Alcaligenes* sp., and *Pseudomonas* sp. from wheat root. Lee et al.^12^ reported N_2_O reduction by the bacterial consortium from the rhizosphere of maize and tall fescue.

**Figure 2:**
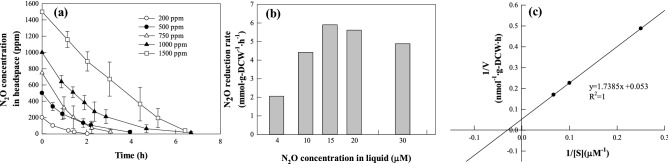
Effect of N_2_O concentration on N_2_O reduction by *Pseudomonas* sp. TF716. (**a**) Time profile of the N_2_O concentration with different initial N_2_O concentrations. (**b**) N_2_O reduction rates at different N_2_O concentrations. (**c**) Lineweaver–Burk plot used to calculate the maximum specific N_2_O reduction rate (*V*_max_).

Figure 2 presents the N_2_O reduction activity of isolate TF716 for different N_2_O loads (200–1500 ppm in the headspace). Isolate TF716 reduced N_2_O without a lag period under the experimental N_2_O loads (Fig. 2a), with the N_2_O reduction rate increasing with higher N_2_O loads until 750 ppm (15 µM in liquid) before slightly decreasing (Fig. 2b). Other studies have also reported that the N_2_O reduction rate increases with the concentration of N_2_O supplied to the final electron acceptor^8,28^. Figure 2c shows the Lineweaver–Burk plot using the data under conditions proportional to the N_2_O reduction rate and the N_2_O concentration (200, 500 and 750 ppm). The maximum N_2_O reduction rate (*V*_*max*_) produced by isolate TF716 was 18.9 mmol N_2_O g dry cells^−1^ h^−1^. In past studies, the N_2_O reduction rate for *Pseudomonas* spp. has been reported to be 2.2–2.6 mmol N_2_O·g dry cells^−1^ h^−1^^8,19,21^, while N_2_O-reducing *Azospira* spp. exhibited a N_2_O reduction rate of 1.60 − 23.85 mmol N_2_O g dry cells^−1^ h^−1^^8,19,22^, and that for *Alicycliphilus denitrificans* was 7.6 mmol N_2_O g dry cells^−1^ h^−1^^22^. Considering these results, the N_2_O-reduction ability of *Pseudomonas* sp. TF716 was superior to that of other N_2_O-reducing bacteria except *Azospira* sp. strain I13 (23.85 mmol N_2_O g dry cells^−1^ h^−1^)^22^.

### Effect of the root exudate on N_2_O-reduction activity

**Figure 3:**
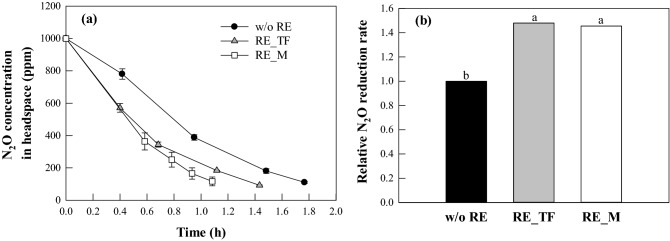
Effect of root exudate (RE) on N_2_O reduction by *Pseudomonas* sp. TF716. **(a)** Time profile of the N_2_O concentration in the medium with or without the RE. (**b**) Comparison of the relative N_2_O reduction rates (w/o RE: without RE; RE_TF: RE of tall fescue; RE_M: RE of maize). Different letters indicate a significant difference in each plot (*p* < 0.05).

Tall fescue and maize are widely used for the rhizoremediation of oil-contaminated soil because their roots are well-developed^29–32^. The interaction between the roots and rhizobacteria is a key parameter for rhizoremediation performance, with the root exudate potentially positively or negatively affecting rhizobacteria activity^33–37^. The effect of tall fescue or maize root exudate on N_2_O reduction by *Pseudomonas* sp. TF716 is presented in Fig. 3. Compared to the control (without root exudate), N_2_O levels were more rapidly reduced when the root exudate was applied. By the addition of the root exudate, the N_2_O reduction rate for strain TF716 increased 1.5-fold of the N_2_O reduction rate at the control (9.9 ± 0.3 mmol N_2_O g dry cells^−1^ h^−1^). There was no significant difference between the exudates for tall fescue (14.7 ± 0.3 mmol N_2_O·g dry cells^−1^ h^−1^) and maize (14.4 ± 0.8 mmol N_2_O·g dry cells^−1^ h^−1^). Greater N_2_O reduction with the addition of root exudate has also been reported in previous studies^12,38^. For example, when root exudate was added to N_2_O-reducing consortia, the N_2_O reduction rate was 1.3–2.7 times higher than without the exudate^12^. The root exudate of plants contains various organic compounds such as sugars, amino acids, organic acids, fatty acids, alcohols, and phosphate^12,39–41^. It is assumed that these organic compounds were utilized as nutrients or growth-promotion factors for strain TF716, thus leading to a higher N_2_O reduction capacity^12,42^.

### Inoculation effect of isolate TF716 on the N_2_O-reduction potential of the soil

Table 1 presents the inoculation effect of isolate TF716 on the N_2_O-reduction potential of diesel-contaminated soil planted with tall fescue for rhizoremediation. In the soil without the inoculation, the N_2_O-reduction potential was 159.4 ± 6.7 nmol g-dry soil^−1^ h^−1^ on day 0, reached its maximum on day 19 (315. ± 17.4 nmol g-dry soil^-1^ h^-1^), and decreased to 279.9 ± 8.6 and 230.6 ± 0.9 nmol g-dry soil^−1^ h^−1^ on days 40 and 59, respectively. This could be because the relative abundance of those bacteria involved in N_2_O reduction increased after the planting of the tall fescue. However, when isolate TF716 was added, the N_2_O-reduction potential ranged from 609.1 ± 32.2 to 760.1 ± 58.9 nmol g-dry soil^−1^ h^−1^ between days 0 and 19. On days 40 and 59, the potential was 304.8 ± 5.1 and 260.3 ± 5.8 nmol g-dry soil^−1^ h^−1^, respectively, half the initial value. This indicates that the population of the inoculated strain remained dominant in the soil for 19 days, but its relative abundance subsequently decreased.

**Table 1:** Comparison of the soil N_2_O-reduction potential during the rhizoremediation of diesel-contaminated soil.

Sampling time (d)	Soil N_2_O-reduction potential (nmol g-dry soil^−1^ h^−1^)			
	TF	TF + 716	M	M + 716
0	159.4 ± 6.7^i^	760.1 ± 58.9^a^	159.4 ± 6.7^i^	760.1 ± 58.9^a^
19	315.1 ± 17.4^c^	609.1 ± 32.2^b^	211.5 ± 11.2^hg^	268.3 ± 15.1^fde^
40	279.9 ± 8.6^cde^	304.8 ± 5.1^cd^	239.5 ± 11.3^feg^	213.9 ± 14.6^hg^
59	230.6 ± 0.9^fg^	260.3 ± 5.8^fdeg^	177.1 ± 1.7^hi^	156.5 ± 2.4^i^

TF: soil planted with tall fescue; TF + 716: soil planted with tall fescue inoculated with strain TF716; M: soil planted with maize; M + 716: soil planted with maize inoculated with strain TF716.

Different letters indicate significant differences between treatments (*p* < 0.05).

When isolate TF716 was added, the N_2_O-reduction potential of the soil was 2.88 times higher than with no inoculation during the initial period (0–19 d) and 1.08–1.13 times thereafter. These results suggest that N_2_O emissions during the rhizoremediation of diesel-contaminated soil using tall fescue can be reduced by adding *Pseudomonas* sp. TF716. Usyskin-Tonne et al.^27^ reported that N_2_O emissions from wheat rhizosphere soil can be reduced by the addition of *Agrobacterium*/*Rhizobium* spp, while *P. stutzeri* PCN-1 can reduce N_2_O emissions during wastewater treatment^43^. In support of this previous research, the present study also demonstrates that the addition of N_2_O-reducing bacteria is a promising strategy to reduce N_2_O emissions during the rhizoremediation of diesel-contaminated soil.

In the soil planted with maize without isolate TF716, the N_2_O-reduction potential increased over the first 40 days from 159.4 ± 6.7 to 239.5 ± 11.3 nmol g-dry soil^−1^ h^−1^, and then it decreased to 177.1 ± 1.7 nmol g-dry soil^−1^ h^−1^ by day 59 (Table 1). This change in the N_2_O-reduction potential with maize was similar to that with tall fescue. When the soil with maize was inoculated with isolate TF716, the soil N_2_O-reduction potential decreased over time from 760.1 ± 58.9 on day 0 to 156.5 ± 2.4 nmol g-dry soil^−1^ h^−1^ on day 59. Thus, with the addition of isolate TF716, the N_2_O-reduction potential increased 4.8- and 1.3-fold on days 0 and 19, respectively, but was slightly lower than without inoculation after 40 days. This indicates that the inoculated strain did not maintain its dominance in the soil planted with maize. Ferrarezi et al.^44^ investigated the inoculation effect of rhizobacteria on maize growth. Depending on the combination of the inoculating rhizobacteria, maize growth was either promoted or inhibited. Thus, when introducing a rhizobacterium, the interaction between the bacterium and the plant roots must be clearly understood to ensure that it is a positive combination.

### Inoculation effect of isolate TF716 on rhizoremediation performance

**Figure 4:**
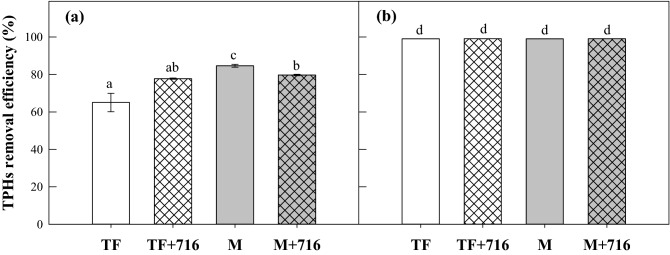
Comparison of the diesel-removal efficiency on days (**a**) 19 and (**b**) 59 during the rhizoremediation of diesel-contaminated soils (TF: soil planted with tall fescue; TF + 716: soil planted with tall fescue with the addition of strain TF716; M: soil planted with maize; M + 716: soil planted with maize with the addition of strain TF716). Different letters indicate a significant difference in each plot (*p* < 0.05).

Although isolate TF716 has no ability to degrade diesel itself (data not shown), the effect of its addition to the soil on diesel degradation was evaluated because other soil microorganisms may be affected by its presence (Fig. 4). In the soil planted with tall fescue, the diesel degradation efficiency in the inoculated soil was not significantly different from that in the non-inoculated soil, although the former was slightly faster than the latter. In the soil planted with maize, the diesel degradation in the non-inoculated soil (85 ± 0.8%) was slightly higher than in the inoculated soil (80 ± 0.4%) on day 19, but there was no difference on day 59. Overall, TF716 inoculation did not affect diesel degradation during rhizoremediation, suggesting that the isolate does not inhibit the activity of the microorganisms that are involved in diesel degradation.

In general, plant growth in contaminated soil is inhibited by pollutants, but it can be improved with the introduction of rhizobacteria. For example, the root growth of castor beans was promoted with the addition of a bacterial consortium into Pb and Zn-contaminated soil^34^. The addition of *Burkholderia phytofirmans* PsJ also reduced the inhibition of the root and stem growth of ryegrass in diesel-contaminated soil^45^, while the inhibition of mangrove in diesel-contaminated soil was reduced by introducing *Bacillus*, *Sphingomonas*, and *Rhodococcus*^46^. In addition, *Gordonia* sp. S2RP-17 enhanced the growth of maize in diesel-contaminated soil^47^, and the shoot and root growth of tall fescue in PCB-contaminated soil increased when the soil was inoculated with *Bulkholeria* spp.^48^.

**Figure 5:**
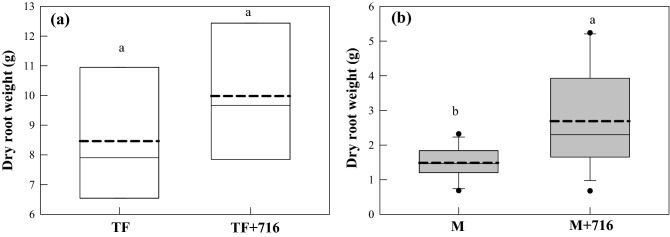
Inoculation effect of *Pseudomonas* sp. TF716 on the root growth of (**a**) tall fescue and (**b**) maize (TF: soil planted with tall fescue; TF + 716: soil planted with tall fescue with the addition of strain TF716; M: soil planted with maize; M + 716: soil planted with maize with the addition of strain TF716). In the box plots, the boxes represent the 25th, 50th, and 75th percentiles, and the error bars indicate the 5th and 95th percentiles. Points outside the boxes indicate outliers. The dashed line indicates the median. Different letters indicate a significant difference in each plot (*p* < 0.05).

To investigate the effect of isolate TF716 on plant growth, its indole-3-acetic acid (IAA)-production ability and 1-aminocyclopropane-1-carboxylic acid (ACC) deaminase activity was first assessed, and it was found that it produced IAA but not ACC deaminase (data not shown). The inoculation effect of the isolate TF716 on the root growth of tall fescue and maize was then evaluated (Fig. 5). Compared with the control groups without inoculation, the addition of strain TF716 slightly enhanced the root growth of tall fescue, although this was not significant. In the soil planted with maize, strain TF716 had little effect on the N_2_O-reduction potential and diesel degradation (Table 1 and Fig. 4), but it unexpectedly enhanced the root growth of maize. Further research is required to elucidate the reason for this.

### Dynamics of the bacterial community associated with N_2_O reduction during the rhizoremediation of diesel-contaminated soil

**Figure 6:**
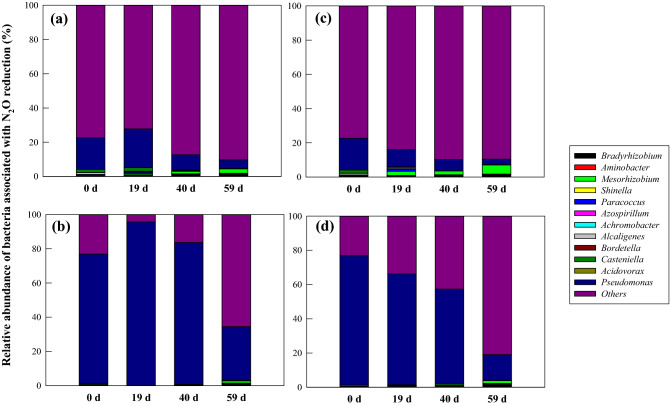
Dynamics of the bacterial genera associated with N_2_O reduction during the rhizoremediation of diesel-contaminated soil. (**a**) Soil planted with tall fescue. (**b**) Soil planted with tall fescue with the addition of strain TF716. (**c**) Soil planted with maize. (**d**) Soil planted with maize with the addition of strain TF716.

Figure 6 shows the dynamics of 12 genera involved in N_2_O reduction during the rhizoremediation of diesel-contaminated soil. Bacterial species belonging to *Bradyrhizobium*, *Aminobacter*, *Mesorhizobium*, *Shinella*, *Paracoccus*, *Azospirillum, Alcaligenes*, *Castellaniella*, and *Pseudomonas* have been reported to reduce N_2_O ^49–61^. Some also contain *nosZ* genes, which are responsible for the reduction of N_2_O to N_2_^52,54,57,59,62–65^. *Bordetella* and *Acidovorax* have been reported to be denitrifying bacteria with *nirS* and *nar/nir* genes, respectively^66–68^.

Based on the analysis of the bacterial community dynamics, the dominant genera in the diesel-contaminated soils were *Pseudomonas*, *Bradyrhizobium*, and *Mesorhizobium*. *Pseudomonas* exhibited the highest relative abundance in all soil samples except for that for the soil planted with maize taken on day 59. In the soil planted with tall fescue, the relative abundance of *Pseudomonas* was 18.5–22.6% for the initial period (0–19 d), decreasing to 9.0% and 5.0% on days 40 and 59, respectively. In the TF716-inoculated soil planted with tall fescue, the relative abundance of *Pseudomonas* increased from 75.5 to 95.5% during the first 19 days, but decreased thereafter to 31.2% on day 59. However, in the soil planted with maize, the relative abundance of *Pseudomonas* tended to decrease with time with or without the presence of TF716. Since the *Pseudomonas* TF716 was isolated from the tall fescue’s rhizosphere, it is thought to be able to grow better in the tall fescue’s rhizosphere compared to maize’s corn rhizosphere. It is estimated that the relative abundance of the genus Pseudomonas to which TF716 belongs has decreased over time because the strain TF716 inoculated into the soil competes with indigenous soil N_2_O-reducers. In all soil samples, the relative abundance of *Bradyrhizobium* and *Mesorhizobium* increased with time, reaching 1.5–2.3% and 1.3–5.3% on day 59, respectively. In contrast, the relative abundance of *Parcoccus*, *Alcaligenes*, and *Catenllaniella* decreased over time.

### Correlation analysis between key parameters

**Figure 7:**
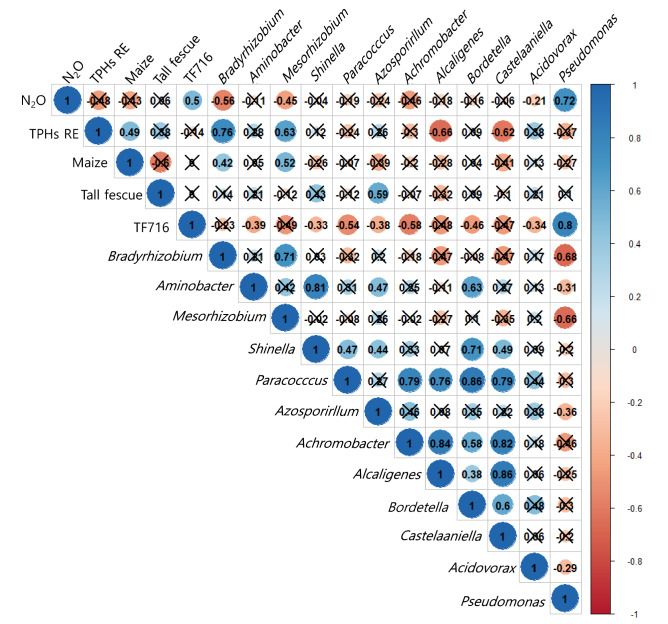
Pearson correlation between key parameters (N_2_O: N_2_O-reduction potential of the soil; TPHs RE: TPH-removal efficiency; Maize: maize planting; Tall fescue: tall fescue planting; TF716: *Pseudomonas* sp. TF716 inoculation; Genus-level bacteria: abundance of bacterial genera).

Figure 7 shows the correlation between TF716 inoculation, the N_2_O-reduction potential of the soil, the TPH-removal efficiency, plant type, and bacterial genera. The N_2_O-reduction potential was positively correlated with the addition of TF716 and the relative abundance of *Pseudomonas* (which includes strain TF716). The N_2_O-reduction potential was positively correlated with the addition of isolate TF716 and the relative abundance of *Pseudomonas*. *Pseudomonas* has previously been utilized as a biosource for the rhizoremediation of TPH-contaminated soil^69–72^. In particular, the introduction of *Pseudomonas*, which has plant growth-promoting traits such as ACC deaminase, IAA, and siderophore production, enhances plant growth, consequently improving remediation performance^69,70^. The positive inoculation effect of *Pseudomonas* in mitigating N_2_O emissions in agricultural soils has also been reported^61,73^. The present study also demonstrates that N_2_O emissions can be reduced during the rhizoremediation of TPH-contaminated soil using *Pseudomonas* TF716.

The TPH-removal efficiency had a close relationship with the relative abundance of *Bradyrhizobium* and *Mesorhizobium*, which were significantly correlated with maize. They also had a positive correlation with each other. Their capacity for N_2_O reduction has been demonstrated in past research^49–51,53,54^. *Bradyrhizobium* can degrade various hydrocarbons such as chloroalkanes, chloroalkenes, and benzonate^74^, and *Mesorhizobium* is a known TPH-degrader^75^. *Bradyrhizobium* and *Mesorhizobium* were also the dominant bacterial groups in *n*-decane-degrading consortia^76^. As shown in Fig. 6, the relative abundance of *Bradyrhizobium* and *Mesorhizobium* increased during the rhizoremediation process. Based on these results, it is clear that they play a major role in TPH degradation and N_2_O reduction in diesel-contaminated soils planted with maize and tall fescue. Although *Azospirillum* is known to promote maize growth^77^, it had a positive correlation with tall fescue but not maize in the present study.

There were also some significant correlations among 12 bacterial genera in the soil samples. *Pseudomonas* sp. TF716 had a negative correlation with most of the bacterial genera. *Aminobacter* had a positive relationship with *Shinella, Azosporillum*, and *Bordetella*, while *Shinella* was positively related to *Paracoccus, Azosporillum*, *Bordetella*, and *Castelaniella*. *Paracoccus* exhibited a high positive correlation with *Achromobacter, Alcaligenes, Bordetella*, and *Castelaniella*. *Achromobacter* was associated with *Bordetella* and *Castelaniella*, while *Alcaligens* had a close relationship with *Bordetella* and *Castelaniella,* and the relative abundance of *Bordetella* was positively related to *Castelaniella*. *Aminobacter* has been reported to degrade 2,6-dichlorobenzamide^78,79^. In addition, *Shinella, Paracoccus*, *Azospirillum*, *Achromobacter*, *Alcaligenes*, *Bordetalla*, *Castenella*, and *Acidovorax* are known to decompose TPHs^75,80–84^. Although no statistical correlation was found, these bacterial groups are assumed to be involved in TPH degradation and nitrogenous compound metabolism, including N_2_O production and mitigation.

## Conclusions

N_2_O is one of the six greenhouse gases listed in the Kyoto Protocol, and it has a GWP that is 298 times higher than that of CO_2_ (IPCC, 2013). During the rhizoremediation of TPH-contaminated soil, nitrogen sources are typically added to promote remediation performance, thus increasing N_2_O emissions. To mitigate N_2_O emissions during the rhizoremediation of TPH-contaminated soil, strategies that effectively reduce these emissions without negatively affecting the TPH-degrading process are required. *Pseudomonas* sp. TF716, isolated from the rhizosphere of tall fescue, exhibited a N_2_O reduction rate of 18.9 mmol N_2_O·g dry cells^−1^ h^−1^, and its N_2_O reduction activity was enhanced by the addition of the root exudate of tall fescue. By introducing *Pseudomonas* sp. TF716 to diesel-contaminated soil planted with tall fescue, the N_2_O-reduction potential of the soil increased without compromising the degradation process. During rhizoremediation for 59 days, *Pseudomonas* maintained the highest relative abundance in the soil. However, no enhancement in the N_2_O-reduction potential of the soil planted with maize was observed when *Pseudomonas* sp. TF716 was added, even though the N_2_O-reduction activity of this bacterium in a liquid medium was improved with the addition of the root exudate from maize. These results indicate that the addition of *Pseudomonas* sp. TF716 is a promising strategy to mitigate N_2_O emissions during the rhizoremediation of diesel-contaminated soil using tall fescue. This study is the first report that it is possible to reduce N_2_O emissions during the rhizoremediation of oil-contaminated soil by the inoculation of a N_2_O-reducing bacterium. Follow-up studies are required to optimize the physicochemical conditions for the activity of *Pseudomonas* sp. TF716 in the contaminated soil. Additionally, further study on the interaction between N_2_O-reducing rhizobacteria and plant roots is needed for the successful mitigation of N_2_O emissions during the rhizoremediation process.

## Supplementary Information

Supplementary Information.

## Data Availability

The sequence for isolate TF716 was deposited in the NCBI GenBank database (http://www.ncbi.nlm.nih.gov/) under accession number MW882239. The obtained sequencing reads for the analysis of the bacterial community were deposited in the NCBI under accession number PRJNA791149.
