# Supplementary material for: Inoculation effect of Pseudomonas sp. TF716 on N2O emissions during rhizoremediation of diesel-contaminated soil
Source: Sci Rep. 2022 Jul 29;12:13018. doi: 10.1038/s41598-022-17356-z (PMC9338077; doi:10.1038/s41598-022-17356-z)
Supplement: Supplementary file 1 — Supplementary Information. [file 41598_2022_17356_MOESM1_ESM.docx]

**[Supplementary materials]**

**Inoculation effect of *Pseudomonas* sp. TF716 on N_2_O emissions during rhizoremediation of diesel-contaminated soil**

Ji-Yoon Kim^1^ · Kyung-Suk Cho^1*^

^1^Department of Environmental Science and Engineering, Ewha Womans University, Seoul 03760, Republic of Korea. ^*^email: kscho@ewha.ac.kr

Table S1 Plant growth-promoting characteristics of *Pseudomonas* sp. TF716

| plant growth-promoting characteristics | Activity | |
| --- | --- | --- |
| IAA synthesis (μg-IAA·mL^-1^) | 49.30±3.182 |  |
| ACC deaminase (OD_600nm_) | 0.038±0.008 |  |

IAA, indole-3-acetic acid;

ACC deaminase, 1-aminocyclopropane-1-carboxylic acid deaminase
